# Supplementary material for: Detecting the ultra low dimensionality of real networks
Source: Nat Commun. 2022 Oct 15;13:6096. doi: 10.1038/s41467-022-33685-z (PMC9569339; doi:10.1038/s41467-022-33685-z)
Supplement: Supplementary file 1 — Supplementary Information [file 41467_2022_33685_MOESM1_ESM.pdf]

Supplementary Information for Detecting the ultra low dimensionality of real networks

Pedro Almagro,[1] Marián Boguñá,[2,3] M. Ángeles Serrano[2,3,4*]

[1]Departamento de Ciencias de la Computación e Inteligencia Artificial,
Universidad de Sevilla,
[2]Departament de Física de la Matèria Condensada, Universitat de Barcelona,
Martí i Franquès 1, 08028 Barcelona, Spain
[3]Universitat de Barcelona Institute of Complex Systems (UBICS),
Universitat de Barcelona, Barcelona, Spain
[4]ICREA, Pg. Lluís Companys 23, E-08010 Barcelona, Spain

*Corresponding author. E-mail: marian.serrano@ub.edu(M. A. S.)

Contents

1 Dimensionality of real networks **3**
1.1 Data description 3
1.2 Estimation of the range of inverse temperatures 6
1.3 Classifier selection 6
1.4 Network features and dimensionality 7
1.5 Phase space of edge cycles in real networks 7

List of Tables

S1 Statistics of the real networks 7

List of Figures

S1 Scaling relation of edge clustering for different dimensions 6
S2 Decision boundaries using different classifiers 6
S3 Relation between cycles and dimensions for real networks 8
S4 Relation between cycles and dimensions for real networks 9
S5 Relation between cycles and dimensions for real networks 10
S6 Relation between cycles and dimensions for real networks 11
S7 Relation between cycles and dimensions for real networks 12
S8 Relation between cycles and dimensions for real networks 13

1 Dimensionality of real networks

We focused on undirected real networks with less than 100K nodes with edge clustering in the range $C_t \in (0.25, 0.8)$ from very different domains as described below.

1.1 Data description

- **AstroPh-CA** [1]: Collaboration network of Arxiv Astro Physics.
- **Bible-CO** [2]: Network containing co-occurrences of nouns (places and names) of the Bible.
- **Cargoships** [3]: Network of global ports connected by commercial lines.
- **CElegans-C** [4]: The nervous systems network of the Caenorhabditis elegans.
- **CondMat-CA** [1]: Collaboration network of Arxiv Condensed Matter.
- **Drosophila-G** [5]: Genetic interactions for Drosophila Melanogaster. We have converted the multiplex network into a single network taking into account the following relationship types: Direct interaction, Suppressive genetic interaction defined by inequality, Additive genetic interaction defined by inequality, Physical association, Colocalization, Association and Synthetic genetic interaction defined by inequality.
- **EnronEmail** [6]: The network of email communication within the Enron company.
- **EUEmail** [7]: The network of email communication in a large European research institution.
- **Facebook-H** [8]: A page-page graph of Facebook sites. Nodes represent Facebook pages and links are mutual likes between sites.
- **Friends-OFF** [9]: A network created from a survey in which each student was asked to list his 5 best female and his 5 male friends. A node represents a student and an edge a between two students shows friendship.
- **Friends-ON** [10]: A network containing friendships between users of the website hamsterster.com.

- **Geom-CA**[11]: The authors collaboration network in computational geometry produced from the BibTeX bibliography. Two authors are linked with an edge, iff they wrote a common work (paper, book, ...).
- **GrQc-CA**[1]: Collaboration network of Arxiv General Relativity.
- **HepTh-CA**[1]: Collaboration network of Arxiv High Energy Physics Theory.
- **HepTh-CIT**[12]: Arxiv High Energy Physics Theory paper citation network.
- **Human-M**[13]: One-mode projection onto metabolites of the human metabolic network at the cell level.
- **Human1-C**[4]: A connectome of the human brain including one hemisphere.
- **Human1-P**[14]: Network of physical protein-protein interaction networks for human ileum tissue. Nodes represent human proteins and edges represent tissue-specific physical interactions between proteins. Original dataset was multi-layer, this network contains only the layer *ileum*.
- **Human2-C**[4]: A connectome of the human brain including one hemisphere.
- **Human2-P**[14]: Network of physical protein-protein interaction networks for human tooth tissue. Nodes represent human proteins and edges represent tissue-specific physical interactions between proteins. Original dataset was multi-layer, this network contains only the layer *tooth*.
- **Human3-C**[4]: A connectome of the human brain including the two hemispheres.
- **Human4-C**[4]: A connectome of the human brain including the two hemispheres.
- **Human5-C**[4]: A connectome of the human brain including the two hemispheres.
- **Human6-C**[15]: A connectome of the human brain including the two hemispheres.
- **Internet**[16]: The network of the Internet at the Autonomous Systems level corresponding to mid 2009.

- **Jazz-CA**[17]: A network of collaborations among jazz musicians and bands that performed between 1912 and 1940.
- **Macaque-C**[18]: A connectome of the macaque cortex.
- **Mouse-C**[19]: A connectome of the mouse brain.
- **Music-CO**[20]: In this network, nodes are chords and connections represent observed transitions among them in a set of songs.
- **PGP-Trust**[21]: Interaction network of users of the Pretty Good Privacy (PGP) algorithm.
- **URVEmail**[22]: Network of e-mail interchanges between members of the Univeristy Rovira i Virgili (Tarragona).
- **USCommute**[23]: This network is based on surveys conducted during the 2000 census, and reflects the daily commuter traffic between US counties. We used the backbone generated in[24].
- **Wiki-H**[25]: The network of Wikipedia pages on editorial norms, in 2015. Nodes are wikipedia entries, and two entries are linked if exists a hyperlink between each other.

1.2 Estimation of the range of inverse temperatures

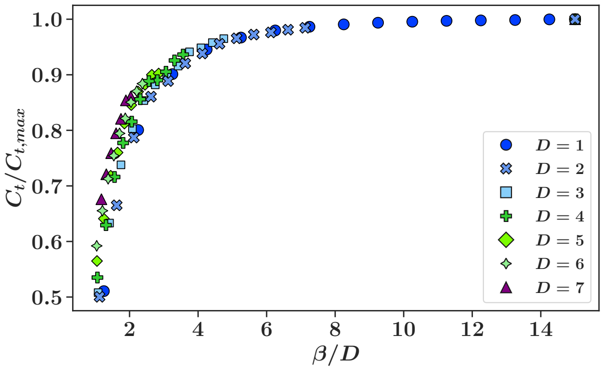

Figure S1: **Scaling relation of edge clustering for different dimensions.** Relation between $C_t/C_{t,max}$ and β/D for a set of networks with the same γ and different values of β and D.

1.3 Classifier selection

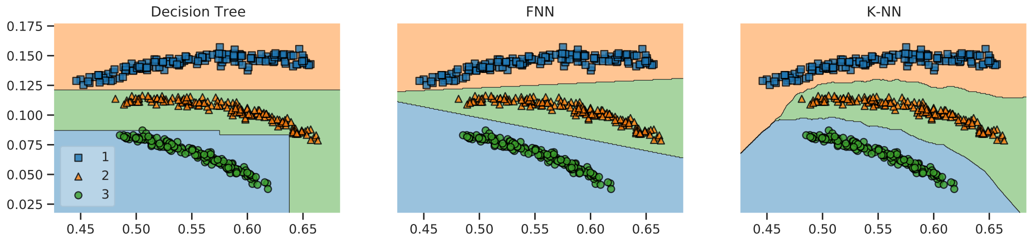

Figure S2: **Decision boundaries using different classifiers.** Examples of decision boundaries when classifying the dimension of a network using a decision tree, a neural network and the k-nearest neighbor model.

1.4 Network features and dimensionality

Table S1 shows the dimension obtained for each real network. In addition, we include properties of every network such as number of nodes/links, average degree and clustering coefficients.

Network	Type	$\|V\|$	$\|E\|$	av. deg.	C	triang	squares	pentag $\times 10^{-3}$	Acc.	D
CElegans-C	Biological - Brain	279	2287	16.3943	0.3371	0.3703	0.1253	2.8196	52.0 %	3
Human1-C	Biological - Brain	493	7773	31.5335	0.4914	0.5443	0.1065	0.7965	100.0 %	3
Human2-C	Biological - Brain	496	8037	32.4073	0.4815	0.5378	0.1061	0.8712	100.0 %	3
Human3-C	Biological - Brain	256	9103	71.1172	0.6839	0.7742	0.1899	0.4983	95.0 %	2
Human4-C	Biological - Brain	360	12100	67.2222	0.6613	0.7427	0.1583	0.4630	100.0 %	2
Human5-C	Biological - Brain	1024	36553	71.3926	0.6002	0.6590	0.1339	0.3695	100.0 %	2
Human6-C	Biological - Brain	989	17865	36.1274	0.4679	0.5194	0.1016	0.7190	100.0 %	3
Macaque-C	Biological - Brain	242	3054	25.2397	0.4501	0.5053	0.1949	1.9376	59.0 %	2
Mouse-C	Biological - Brain	213	2969	27.8779	0.4498	0.5121	0.1930	1.8983	78.0 %	1
Drosophila-G	Biological - Cell	8114	38909	9.5906	0.1000	0.1963	0.0371	0.2907	66.0 %	8
Human-M	Biological - Cell	1436	4718	6.5710	0.5112	0.5866	0.0770	1.1356	85.0 %	3
Human1-P	Biological - Cell	913	7472	16.3680	0.3624	0.2956	0.0970	1.4879	51.0 %	3
Human2-P	Biological - Cell	1090	9369	17.1908	0.3356	0.2448	0.0827	1.3384	53.0 %	1
Facebook-H	Citation - Hyperlinks	22470	170823	15.2045	0.3633	0.4933	0.1083	0.6672	98.0 %	1
Wiki-H	Citation - Hyperlinks	1872	15367	16.4177	0.3828	0.4265	0.1016	0.7636	69.0 %	5
HepTh-CIT	Citation - Scientific	27400	352021	25.6950	0.2355	0.3558	0.0735	0.3389	96.0 %	4
Jazz-CA	Collaboration - Music	199	2907	29.2161	0.6517	0.7472	0.1219	1.0064	100.0 %	2
AstroPh-CA	Collaboration - Scientific	17903	196972	22.0044	0.6332	0.6284	0.0101	0.0769	100.0 %	3
CondMat-CA	Collaboration - Scientific	21363	91286	8.5462	0.6420	0.6808	0.0045	0.0670	99.0 %	3
Geom-CA	Collaboration - Scientific	3621	9461	5.2256	0.5398	0.6830	0.0102	0.2827	100.0 %	3
GrQc-CA	Collaboration - Scientific	4158	13422	6.4560	0.5572	0.7634	0.0053	0.1656	100.0 %	1
HepTh-CA	Collaboration - Scientific	8638	24806	5.7435	0.4820	0.5721	0.0091	0.2753	100.0 %	4
Bible-CO	Coocurrences - Language	1707	9059	10.6139	0.7100	0.6259	0.0347	0.5353	98.0 %	5
Music-CO	Coocurrences - Music	2476	20624	16.6591	0.6756	0.7781	0.0659	0.2250	62.0 %	6
Friends-OFF	Social Offline - Friends	2539	10455	8.2355	0.1467	0.1785	0.0282	1.2503	69.0 %	9
EUEmail	Social Online - Email	986	16064	32.5842	0.4071	0.4735	0.1363	0.8990	89.0 %	3
EnronEmail	Social Online - Email	33696	180811	10.7319	0.5092	0.5434	0.0401	0.1581	95.0 %	8
URVEmail	Social Online - Email	1133	5451	9.6222	0.2202	0.2836	0.0608	1.6337	69.0 %	6
Friends-ON	Social Online - Friends	2000	16098	16.0980	0.5401	0.5241	0.0543	0.6532	99.0 %	6
PGP-Trust	Social Online - Trust	10680	24316	4.5536	0.2659	0.6185	0.0765	0.8444	99.0 %	1
Internet	Technological - peer2customer	23748	58414	4.9195	0.3604	0.5784	0.0534	0.2150	66.0 %	7
USCommute	Transport - Commute	3025	6602	4.3650	0.3678	0.5018	0.0470	3.3138	99.0 %	4
Cargoships	Transport - Ships	821	4342	10.5773	0.4236	0.5482	0.0873	1.4362	85.0 %	3

Table S1: Properties of real networks and their dimensionality as inferred by our method. Notice that we always work with the giant connected component.

1.5 Phase space of edge cycles in real networks

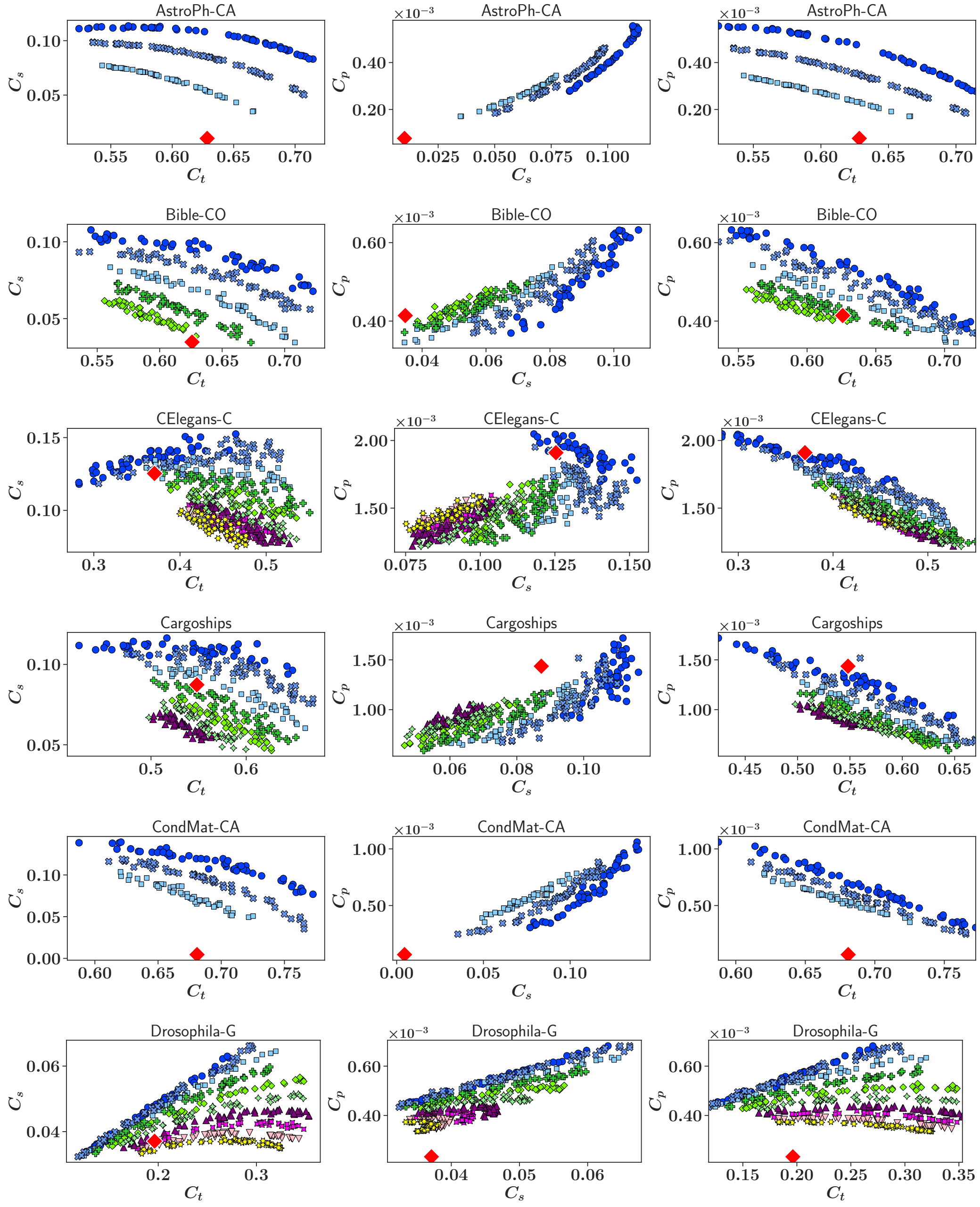

Figure S3: **Relation between cycles and dimensions for real networks.** In each row, and from left to right, graphs show the projection of the phase space in the subspaces (C_s, C_t), (C_p, C_t), and (C_p, C_s). Each point represents an average over 10 network realizations. Standard errors are smaller than the symbols themselves. Every row corresponds to a different real network as indicated in the label above each graph.

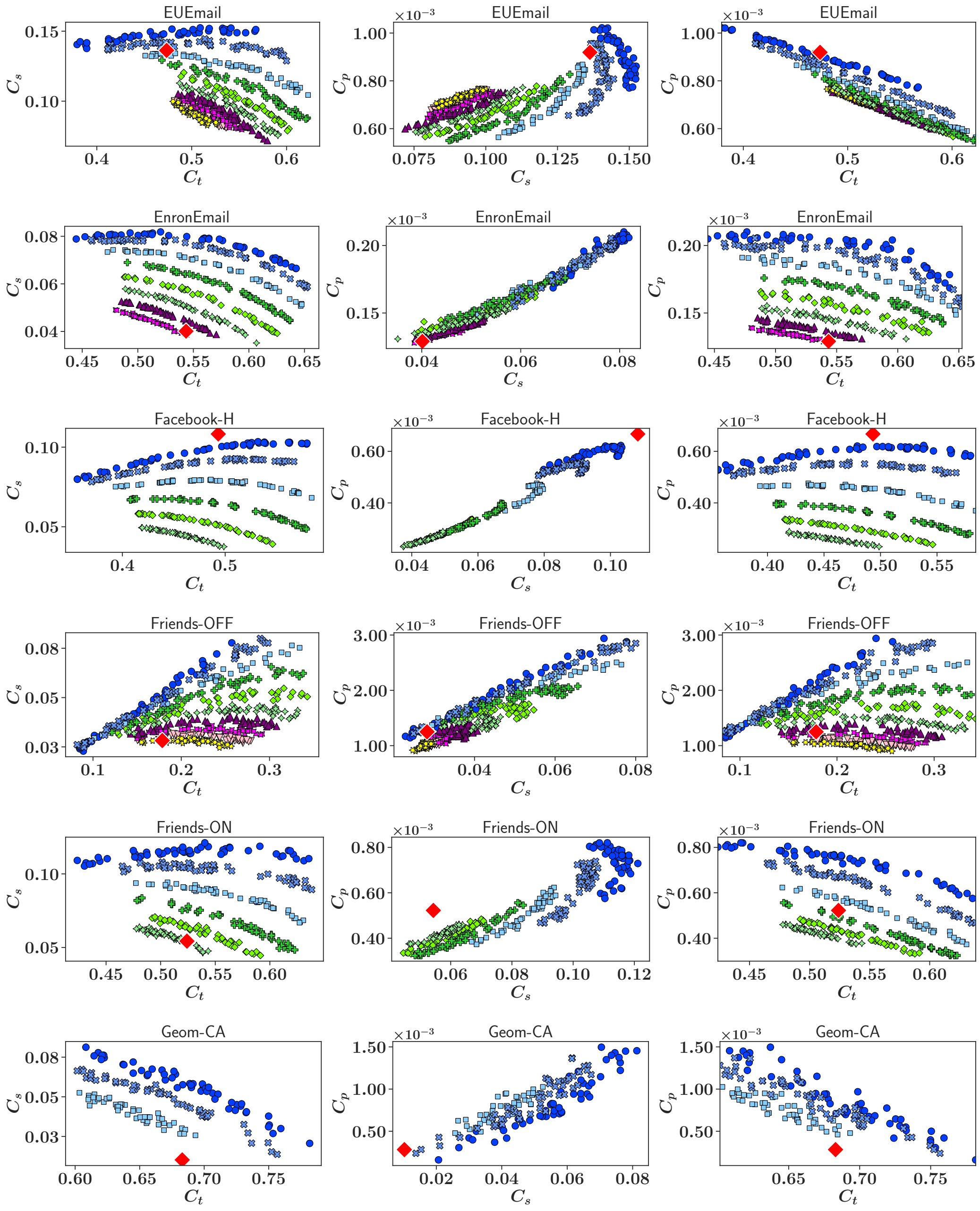

Figure S4: **Relation between cycles and dimensions for real networks.** In each row, and from left to right, graphs show the projection of the phase space in the subspaces (C_s, C_t), (C_p, C_t), and (C_p, C_s). Each point represents an average over 10 network realizations. Standard errors are smaller than the symbols themselves. Every row corresponds to a different real network as indicated in the label above each graph.

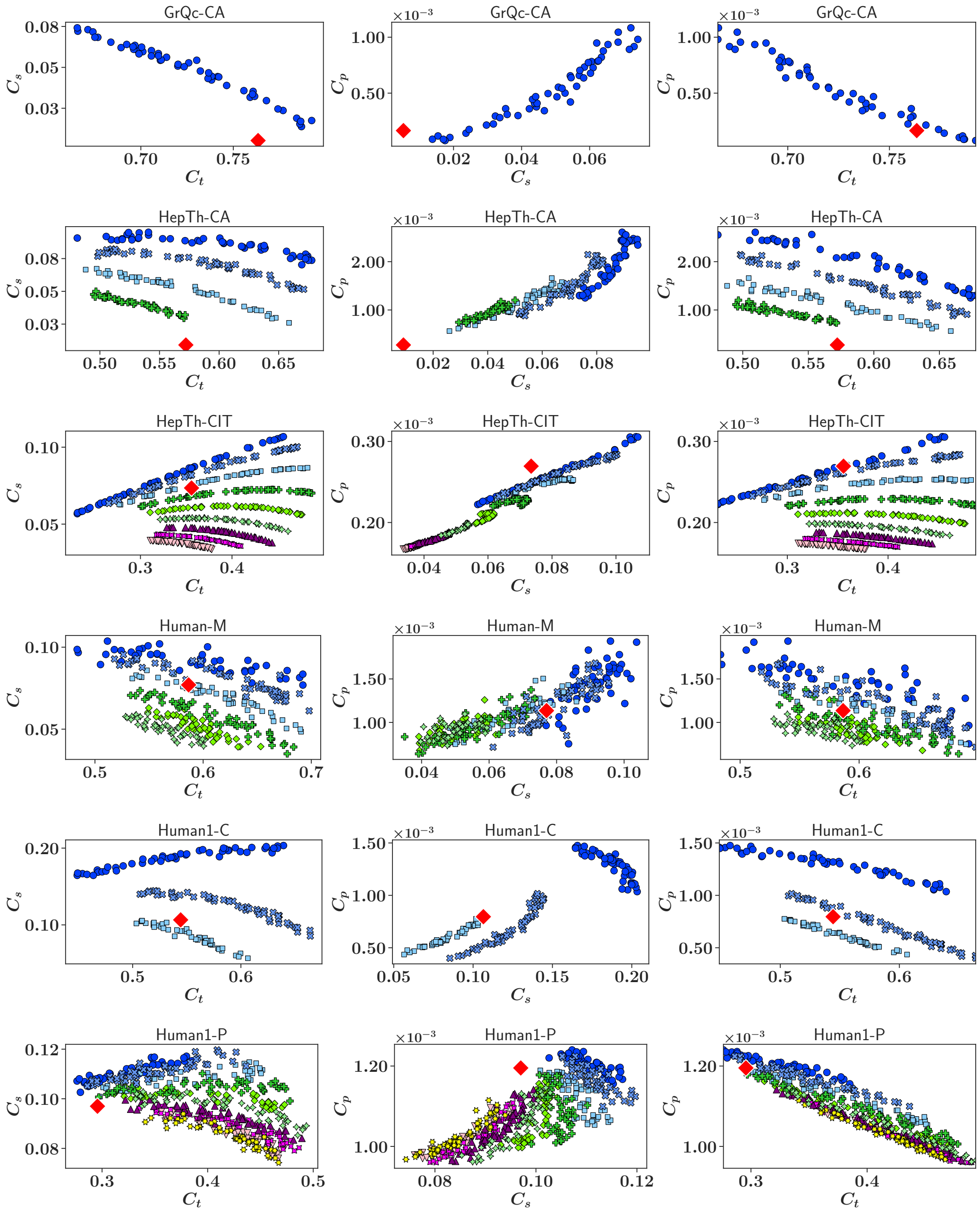

Figure S5: **Relation between cycles and dimensions for real networks.** In each row, and from left to right, graphs show the projection of the phase space in the subspaces (C_s, C_t), (C_p, C_t), and (C_p, C_s). Each point represents an average over 10 network realizations. Standard errors are smaller than the symbols themselves. Every row corresponds to a different real network as indicated in the label above each graph.

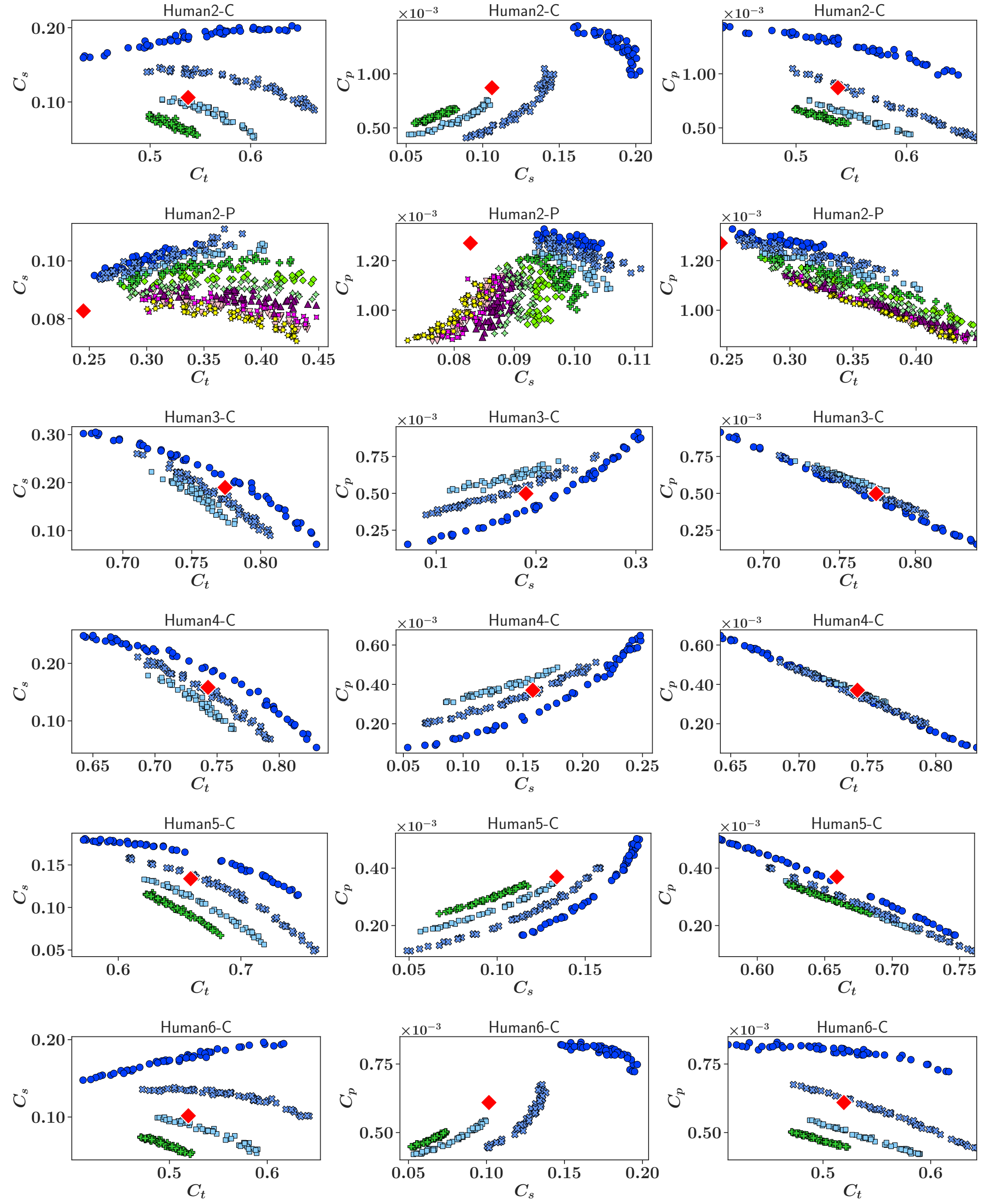

Figure S6: **Relation between cycles and dimensions for real networks.** In each row, and from left to right, graphs show the projection of the phase space in the subspaces (C_s, C_t), (C_p, C_t), and (C_p, C_s). Each point represents an average over 10 network realizations. Standard errors are smaller than the symbols themselves. Every row corresponds to a different real network as indicated in the label above each graph.

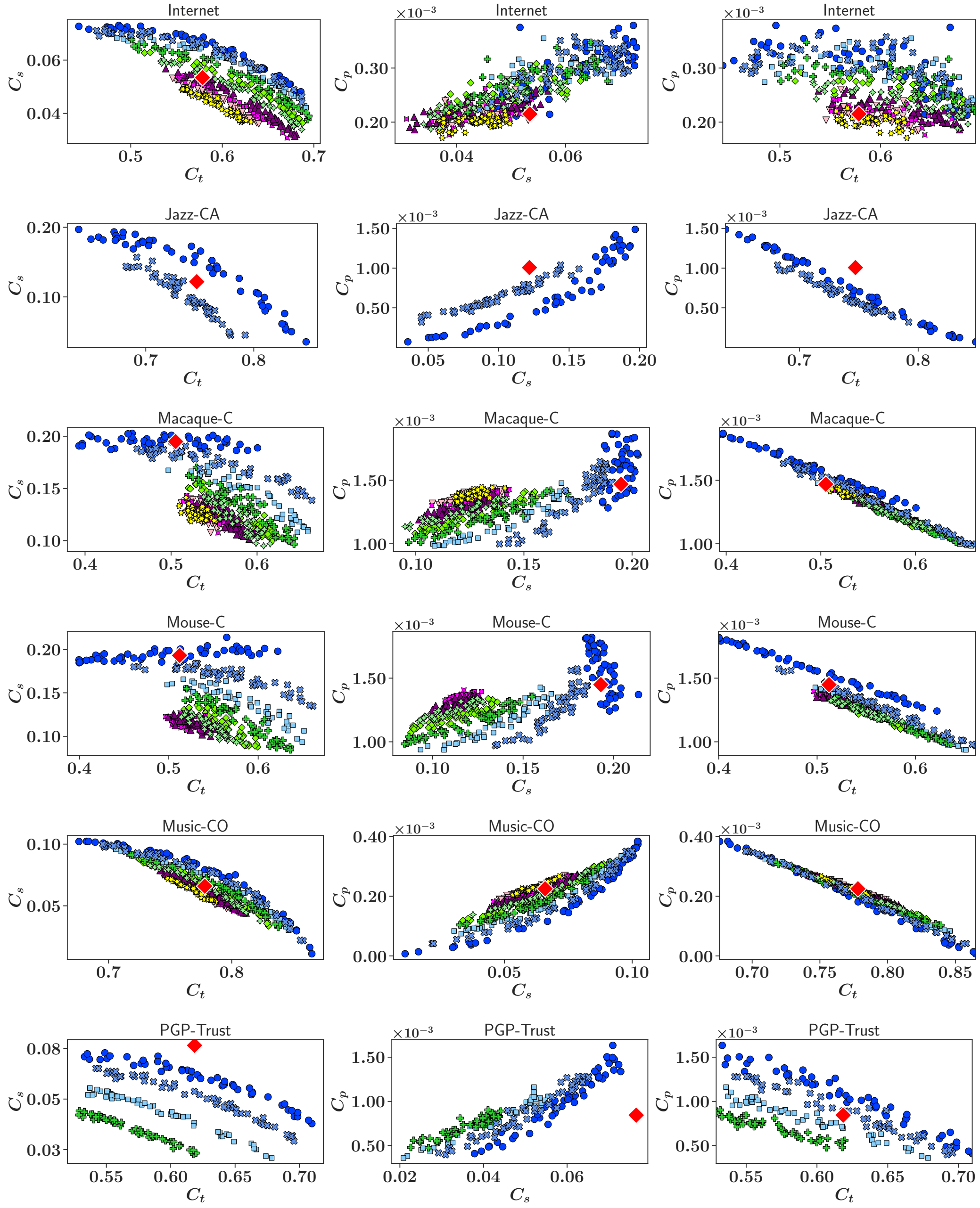

Figure S7: **Relation between cycles and dimensions for real networks.** In each row, and from left to right, graphs show the projection of the phase space in the subspaces (C_s, C_t), (C_p, C_t), and (C_p, C_s). Each point represents an average over 10 network realizations. Standard errors are smaller than the symbols themselves. Every row corresponds to a different real network as indicated in the label above each graph.

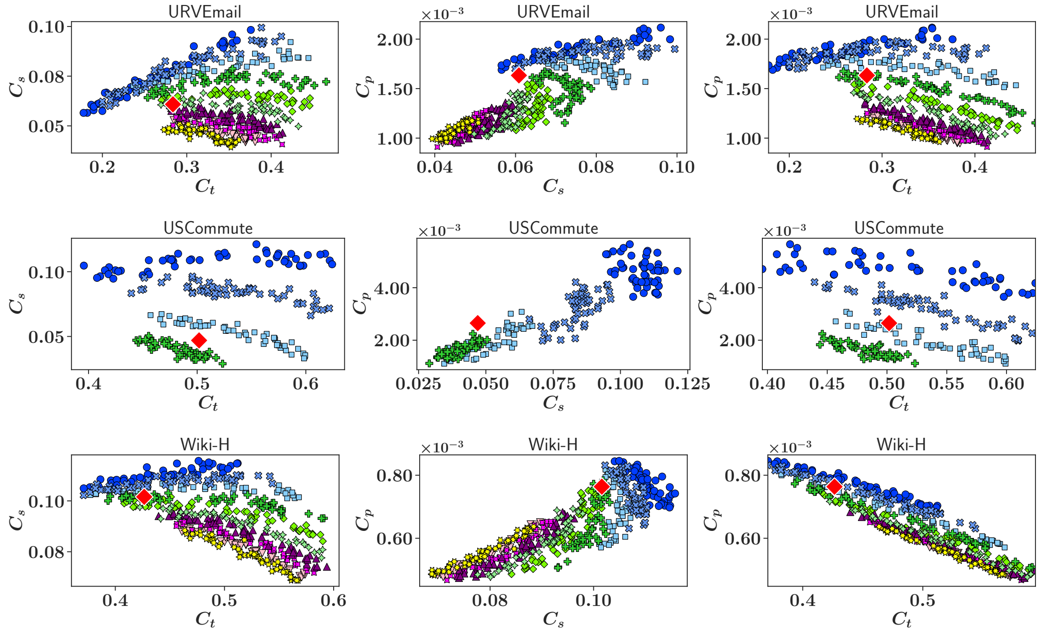

Figure S8: **Relation between cycles and dimensions for real networks**. In each row, and from left to right, graphs show the projection of the phase space in the subspaces (C_s, C_t), (C_p, C_t), and (C_p, C_s). Each point represents an average over 10 network realizations. Standard errors are smaller than the symbols themselves. Every row corresponds to a different real network as indicated in the label above each graph.

Supplementary References

1. Jure Leskovec, Jon Kleinberg, and Christos Faloutsos. Graph evolution: Densification and shrinking diameters. *ACM transactions on Knowledge Discovery from Data (TKDD)* **1**(1), 2–es (2007).

2. `http://chrisharrison.net/projects/bibleviz/index.html`. Accessed: 2014-08-22.

3. Pablo Kaluza, Andrea Kölzsch, Michael T Gastner, and Bernd Blasius. The complex network of global cargo ship movements. *Journal of the Royal Society Interface* **7**(48), 1093–1103 (2010).

4. Yong-Yeol Ahn, Hawoong Jeong, and Beom Jun Kim. Wiring cost in the organization of a biological neuronal network. *Physica A: Statistical Mechanics and its Applications* **367**, 531–537 (2006).

5. Chris Stark, Bobby-Joe Breitkreutz, Teresa Reguly, Lorrie Boucher, Ashton Breitkreutz, and Mike Tyers. Biogrid: a general repository for interaction datasets. *Nucleic acids research* **34**(suppl_1), D535–D539 (2006).

6. Bryan Klimt and Yiming Yang. The enron corpus: A new dataset for email classification research. In Machine Learning: ECML 2004, Jean-François Boulicaut, Floriana Esposito, Fosca Giannotti, and Dino Pedreschi, editors, 217–226 (Springer Berlin Heidelberg, Berlin, Heidelberg, 2004).

7. Hao Yin, Austin R Benson, Jure Leskovec, and David F Gleich. Local higher-order graph clustering. In *Proceedings of the 23rd ACM SIGKDD International Conference on Knowledge Discovery and Data Mining*, 555–564, (2017).

8. Benedek Rozemberczki, Carl Allen, and Rik Sarkar. Multi-Scale attributed node embedding. *Journal of Complex Networks* **9**(2), 05 (2021). cnab014.

9. James Moody. Peer influence groups: Identifying dense clusters in large networks. *Social Networks* **23**(4), 261–283 (2001).

10. Jérôme Kunegis. Konect: the koblenz network collection. In *WWW 2013 Companion - Proceedings of the 22nd International Conference on World Wide Web*, 1343–1350, (2013).

11. N. H. F. Beebe. http://www.math.utah.edu/ beebe/bibliographies.html.

12. Johannes Gehrke, Paul Ginsparg, and Jon Kleinberg. Overview of the 2003 kdd cup. *Acm Sigkdd Explorations Newsletter* **5**(2), 149–151 (2003).

13. M Ángeles Serrano, Marián Boguná, and Francesc Sagués. Uncovering the hidden geometry behind metabolic networks. *Molecular biosystems* **8**(3), 843–850 (2012).

14. Antje Chang, Ida Schomburg, Sandra Placzek, Lisa Jeske, Marcus Ulbrich, Mei Xiao, Christoph W Sensen, and Dietmar Schomburg. Brenda in 2015: exciting developments in its 25th year of existence. *Nucleic acids research* **43**(D1), D439–D446 (2015).

15. Patric Hagmann, Leila Cammoun, Xavier Gigandet, Reto Meuli, Christopher J Honey, Van J Wedeen, and Olaf Sporns. Mapping the structural core of human cerebral cortex. *PLoS Biol* **6**(7), e159 (2008).

16. Kimberly Claffy, Young Hyun, Ken Keys, Marina Fomenkov, and Dmitri Krioukov. Internet mapping: from art to science. In *2009 Cybersecurity Applications & Technology Conference for Homeland Security*, 205–211. IEEE, (2009).

17. Pablo M Gleiser and Leon Danon. Community structure in jazz. *Advances in complex systems* **6**(04), 565–573 (2003).

18. Logan Harriger, Martijn P Van Den Heuvel, and Olaf Sporns. Rich club organization of macaque cerebral cortex and its role in network communication. *PloS one* **7**(9) (2012).

19. SW Oh, JA Harris, L Ng, B Winslow, N Cain, S Mihalas, Q Wang, C Lau, L Kuan, AM Henry, et al. A mesoscale connectome of the mouse brain. *Nature* **508**, 207–214 (2014).

20. Joan Serrà, Álvaro Corral, Marián Boguñá, Martín Haro, and Josep Ll Arcos. Measuring the evolution of contemporary western popular music. *Scientific reports* **2**, 521 (2012).

21. Marián Boguñá, Romualdo Pastor-Satorras, Albert Díaz-Guilera, and Alex Arenas. Models of social networks based on social distance attachment. *Phys. Rev. E* **70**(5), 056122 (2004).

22. Roger Guimerà Manrique, Leon Danon, Albert Díaz Guilera, Francesc Giralt, and Àlex Arenas. Self-similar community structure in a network of human interactions. *Physical Review E* **68**, 065103 (2003).

23. Daniel Grady, Christian Thiemann, and Dirk Brockmann. Robust classification of salient links in complex networks. *Nature communications* **3**(1), 1–10 (2012).

24. Antoine Allard, M. Ángeles Serrano, Guillermo García-Pérez, and Marián Boguñá. The geometric nature of weights in real complex networks. *Nat Commun* **8**, 14103 (2017).

25. Bradi Heaberlin and Simon DeDeo. The evolution of wikipedia's norm network. *Future Internet* **8**(2), 14 (2016).
